# Smoky Chimneys

**Published:** 1866-03

**Authors:** 


### SMOKY CHIMNEYS.

This household calamity can easily be prevented, and always in building new houses; thus, let the throat of the chimney be so constructed that immediately inside of it, the space shall be abruptly increased several inches in length and breadth; let it increase upward for two or three feet, and then be gradually "drawn in" to the dimensions necessary, and let the whole inside of the chimney be plastered with cement, which will harden with time. A very convenient method of ventilating a room already built, is to arrange that one of the "panes" of glass at the upper edge of the sash shall move on a pivot at the center of each side, so that it can be turned, the upper end outward, the lower end inward, or *vice versa;* or to prevent breakage, a thin board painted white, or a piece of tin or zinc may be made to replace the glass. A similar arrangement in new houses will have its conveniences. But in every room this device should be near the ceiling, above the fireplace; for ordinary rooms, the orifice should be a foot long and five or ten inches broad, and arranged so that a cord shall open or close it, without the necessity of getting on a chair or step-ladder.

In building a house in the country, it will save expense and trouble, besides preparing the way for a great deal of comfort on emergencies, to have a neat opening left for a stove-pipe near the ceiling, in at least one room in the house, say the dining-room or parlor, so that in case of excessive cold weather, a common stove for burning wood (or coal) may be put up, and thus have the facilities of making at least one room in the house comfortably warm during any "spell" of bitter cold weather; and warmed, too, at a comparatively small expense. For let it be remembered, that with a common fireplace or grate, more than one half the heat goes up the chimney, and is an utter waste. The longer a stove-pipe is, the more heat is saved in the room; hence the advantage of having the arrangement for receiving the stove-pipe near the ceiling. Many persons, for the sake of appearances, or from a mistaken notion of economy as to the cost of pipe, have the pipe adjusted so as to open into the fireplace, by which a very large amount of heat is lost.

Much has been said of the injurious effects of a dry stove air, and to obviate this, it has been recommended that a vessel of water be kept standing on the stove. If this is left to be attended to by servants, it is far better to have nothing of the kind, because unless the pan is of white stoneware, and is emptied, washed, and filled with pure, fresh water every three or four hours, it "collects" dust, dirt, gases, and emanations, which, by being kept warm, generate a most pernicious malaria, which is much more likely to produce disease than a simple dry air.

It should be remembered that a room is but very little ventilated, and even that very slowly, by simply opening a door or folding-doors. Many persons ignorantly, and to their own injury, rely upon this method of ventilation, when they sleep in the same room in which a fire has been kept all day; and for this reason, also, every chamber should have a ventilator arranged in the original construction of the house.

The coolest part of a room in warm weather, for sleeping, is the floor; but by the operation of the same law of nature, that cool air is heavy, and falls to the surface, the healthiest part of a chamber in very cold weather is the higher. A sleeping person consumes two hogsheads of air in an hour; that is, deprives it of all its oxygen, and replaces it with carbonic acid gas, which is a negative poison; leaving it so destitute of any life-giving property, that the person breathing it will die in a short time—in an hour sometimes. This is the operation going on in a close room where charcoal is burning in an open vessel; the oxygen is consumed in burning the coal, and its place is supplied by carbonic acid. Cold condenses this carbonic acid makes it heavy, and causes it to "settle" on the floor. It has been so condensed by cold as to be made visible in the shape of a snow-white substance; just as the invisible warm moist air, by the application of cold, is reduced to mist, to dew, to rain-drops, and to solid hail-stones. There are some localities in Italy and elsewhere, into which if a man and his dog come, the dog will die in a minute or two, while his master will remain uninjured. There was carbonic acid there; it was concentrated, condensed, made heavy, and settled on the surface, where the dog breathed it; but the man's nostrils being five or six feet higher, took in none of it. From these facts, two practical lessons of very great importance to human health and life are drawn:

First. There is more need of ventilating a chamber in winter than in summer.

Second. There is no advantage, as to health, in sleeping in a very cold room, cold enough to have ice formed in it during the night. Thousands of persons who have gone to bed in perfect health at night, have waked up next morning with "pneumonia," that is, inflammation of the lungs, and have died in a few days, because the room was too cold for them; to say nothing of the debilitating effect of breathing an atmosphere more or less loaded with carbonic acid gas, which deprived the system of its ability to resist the approach of disease. Had the room been well ventilated, the attack would have been less severe, or there might have been none at all; because the breathing of a pure air would have given power to ward off any ordinary attack of sickness. Hence there are the most conclusive reasons for building houses, or remodeling them, so as to have the utmost facilities for ventilation.

Really, every chamber should have two systems of ventilation, internal and external, so that either may be employed, according to the season of the year, and the health and vigor or peculiarity of the sleepers — the internal ventilation, that is, openings above the fireplaces, for feeble persons, or for very cold weather, or in the autumn; the external, that is, through the windows, from all out-doors, for the vigorous, and in moderate weather.

To some persons, in any latitude, and to all in some sections of the country, it is certain suffering to sleep with an open window, especially in August and September; and by understanding the reason of this fully, the necessity may be removed from some families of "selling out," or of building elsewhere. Before changing a residence on account of its being unhealthful, it should first be noticed whether it is connected with any special season of the year, with any special part of the house, or any particular habit of the persons who are attacked; in other words:

Does the sickness appear during the autumnal months?

Does it appear among that part of the family sleeping on the same side of the house; on the northern side, for example, keeping the rooms always more or less damp; or in that part of the building nearest to some pond, or marsh, or sluggish stream; or whether, of several persons sleeping on the same side, only those are attacked who sleep with their windows open?

As a general rule, young children, invalids, infirm and old people, should have their chambers, during the night, ventilated from within; and so should all families living in "bottoms," on low lands, near ponds, sluggish streams, marshes, or recently cleared land, especially during the autumnal months, or where there is more or less of chill and fever, fever and ague, etc. The reason for this is, that from these localities miasm constantly rises and comes through the open windows upon the sleeper, who breathes it into his lungs, corrupting and poisoning his whole blood in a night!

Many cases are given in standard medical publications, where persons sleeping in certain parts of a building suddenly became ill, although they formerly had good health, and had occupied the same chambers, and had slept with open windows all the time; but a change of dwelling, or a determination to build elsewhere, should not be hastily made by the farmer, for some standing water may have been drawn off recently for a merely temporary purpose, the repairing of a mill-dam, for example; and when reflooded, so as to cover the wet, muddy bottom several feet deep in water, the sickness will immediately disappear; or a "belt" of timber between the dwelling and some standing, miasm-producing water, may have been cut down; if so, a substitute should be provided, by planting a thick hedge of sun-flowers or other rapidly-growing and luxuriant vegetation.

THE LOWER FLOOR of every country house should be on the same level; for every step upward taken by domestics and women in the family, is not only a useless expenditure of strength, and a large portion of it too, when it is considered how many times in a day the cook and housemaid and wives and daughters who do the household work must go in and out, and pass and repass from one room to another; but it is physiologically a great strain upon those internal organs which are peculiar to the sex; and when too much of it is done, diseases are every day induced which are to embitter the whole after-existence. It is very easy to wink the eye—an inappreciable effort—but if a man attempts to do it a hundred times in succession, its repetition becomes a painful effort. It is very easy to step up a step or two, but the strongest will "pant and blow" if a hundred have to be gone up, as briskly as an ordinary cook steps about. It may be said that the objection does not apply, because only one step is taken at a time; but it must be remembered that those who do housework almost always have something in the hand—a bucket of water, a pile of plates, an armful of wood, a scuttle of coal, etc., and these must be raised that one step, besides the body of the person, altogether weighing between one and two hundred pounds. A certain amount of strength is expended in this unnecessary effort, and however small it is, each repetition of it is that much taken from the store of strength with which the person arose in the morning. A purse containing a hundred dollars is as much depleted by taking out a dollar at a time, until fifty are withdrawn, as if the whole fifty were detracted at once.

The kitchen should, as far as practicable, be central to the whole house, having the dining-room on one side, the wood-house on another, and the place for meats, milk, and vegetables on another, unless these are all kept in the cellar, located as previously advised. If, however, the dairy is an important item about the farm, that is, if it is intended as a source of income, it should be arranged by all means to be on the north side of a hill or rising ground, and in such a way that a natural stream should flow through it, or that the surplus water of the well or spring or cistern should do so; but by all means let the dairy be approached from the kitchen by a raised graveled walk, with a view to have it as dry as possible at all seasons; for this walk must be passed over many times every day, and if not dry, it dampens the feet, and thus endangers the health.

